# Video Games Exposure and Sexism in a Representative Sample of Adolescents

**DOI:** 10.3389/fpsyg.2017.00466

**Published:** 2017-03-31

**Authors:** Laurent Bègue, Elisa Sarda, Douglas A. Gentile, Clementine Bry, Sebastian Roché

**Affiliations:** ^1^LIP-PC2S, Université Grenoble AlpesGrenoble, France; ^2^Department of Psychology, Iowa State University, AmesLA, USA; ^3^LIP-PC2S, Université Savoie Mont BlancChambéry, France; ^4^CNRS, PACTE, Université Grenoble AlpesGrenoble, France

**Keywords:** sexism, gender role, video games, television, religiosity

## Abstract

Research has indicated that many video games are saturated with stereotypes of women and that these contents may cultivate sexism. The purpose of this study was to assess the relationship between video game exposure and sexism for the first time in a large and representative sample. Our aim was also to measure the strength of this association when two other significant and well-studied sources of sexism, television exposure and religiosity, were also included in a multivariate model. A representative sample of 13520 French youth aged 11–19 years completed a survey measuring weekly video game and television exposure, religiosity, and sexist attitudes toward women. Controlling for gender and socioeconomic level, results showed that video game exposure and religiosity were both related to sexism. Implications of these results for future research on sexism in video games are discussed.

## Introduction

The media are a powerful socializing agent of the modern era. Video games represent one of the most popular forms of media entertainment around the world, with a global market of more than 90 billion dollars in 2015. Part of the popularity may be due to the appeal of masculinity: sales are highest in teen and mature games with box art depicting non-central sexualized female characters (Near, 2013). The depiction and value granted to women is biased in traditional forms of media such as children’s books, magazines and TV (Signorielli and Bacue, 1999; Scharrer, 2014), and there is no obvious exception with new digital media. It has even been argued that some of the most blatantly sexist representation of women is found today in video games (Dill and Thill, 2007; Downs and Smith, 2010; Scharrer, 2014). However, while the proofs of biased depictions in video games showing women as passive beings, kidnapped princess to rescue or sex objects to win or to use are numerous and indisputably recorded (Provenzo, 1991; Beasley and Collins Standley, 2002; Burgess et al., 2007; Dill and Thill, 2007; Near, 2013), their effect on gamers’ stereotypes of women remains debated (Breuer et al., 2015) despite some preliminary experimental demonstrations (Dill et al., 2008; Behm-Morawitz and Mastro, 2009; Fox and Bailenson, 2009; Yao et al., 2010; Driesmans et al., 2015; Gabbiadini et al., 2016).

The present study used a large representative sample of adolescents to analyze the link between video game exposure and the endorsement of sexist attitudes toward women. We also compare this link to two other consequential and well-studied influences on sexist attitudes. The first is television exposure, which has been shown as a major factor involved in sexist depiction of females (Herrett-Skjellum and Allen, 1996; Morgan and Shanahan, 1997; Signorielli and Bacue, 1999; Oppliger, 2007). The other important factor is religiosity, which has a significant influence on stereotyped gender role beliefs (Wilson, 1978; Morgan, 1987; Kirkpatrick, 1993; Hunsberger et al., 1999; Glick et al., 2002; Meghan Burn and Busso, 2005; see, however, Read, 2003). The introduction of these influential additional determinants of sexism will provide us with the opportunity to have a more precise and embedded view of the specific relationship between video games exposure and sexism among adolescents.

### Representation of Gender in Video Game

Since their origins, video games have tended to portray women as characters needing help or holding passive or instrumental roles (Downs and Smith, 2010; Near, 2013; Stermer and Burkley, 2015). In video game magazines, over 80% of female characters are portrayed according to three types: sexualized, scantily clad, or vision of beauty, and over a quarter fit in all three categories (Dill and Thill, 2007). While it appears that women are generally underrepresented in video games (Scharrer, 2004; Dill et al., 2005; Williams et al., 2009; Downs and Smith, 2010), they are frequently presented as attractive beings (Scharrer, 2004), sex objects (Dietz, 1998; Burgess et al., 2007; Jansz and Martis, 2007; Downs and Smith, 2010) and in sexually suggestive ways (Ivory, 2006). In one of the first extensive content analyses of top selling games, Dietz (1998) showed that in over a quarter of games women were depicted as sex objects. Other more recent content analysis showed that women are often displayed with revealing clothing or at least partially nude (Beasley and Collins Standley, 2002; Miller and Summers, 2007; Downs and Smith, 2010). For example, in a systematic analysis of 47 randomly selected games, female characters were more likely to wear low-cut clothing and bare arms than males. Of the 71 female characters for whom cleavage could be seen on, 2.82%, were considered “flat” by independent judges, 56.34%, were considered “average,” and 40.85%, were considered “voluptuous” (Beasley and Collins Standley, 2002). Moreover, while male characters have generally normal sizes in video games (compared to actual sizes of adults), female characters are depicted as thinner (Martins et al., 2011).

According to Bandura’s Social Cognitive Theory (Bandura, 2001), symbolic representations of the world are learned through exposure to models. People rely on such acquired knowledge structures to perceive others and interact with them. Developments in media psychology suggest that the digital representation of the female body is not a mere innocent succession of entertaining polychromatic polygons on a screen, but can change users’ attitudes and behaviors off-screen (Yee and Bailenson, 2007; Fox and Bailenson, 2009; Blascovitch and Mc Call, 2014). Previous theoretical developments suggest that users learn long-term schemas about gender, and that such schemas influence everyday interactions in potentially detrimental ways for women. According to the cultivation theory (Gerbner et al., 1986), repeated exposure to media content influences how social realities are perceived and understood. Adolescents who play video games may model beliefs about gender role on the distorted reality presented in video games. Relevant to the present study, given that a high percentage of video games include sexist and even misogynistic portrayals of females, we hypothesize that more spent time playing video games should predict higher sexist attitudes.

Because video games are designed for iterative play (potentially more than 100 h for the more developed products) and are inherently interactive, they enable repeated and distributed learning about gender roles that may have enduring effects. Video game characters can be agents of gender socialization among youth, and the amount of exposure to video games should predict the endorsement of gender stereotypes. Some studies have supported this prediction. Several experimental studies showed the short-term effect of sexualised video games on sexist attitudes. These studies show that playing sexualized video games for a few minutes (10–20 min) promotes men’s likelihood to endorse gender stereotypes (Behm-Morawitz and Mastro, 2009; Yao et al., 2010), increase hostile sexism (Fox and Bailenson, 2009), and increases men’s acceptance of sexual harassment (Dill et al., 2008).

Fewer studies have been interested in long-term links between video game and sexist attitudes. In one cross-sectional study, it was shown that men who regularly played video games with sexist contents (according to their own evaluation of sexism) tended to have a higher level of benevolent sexism, that is associated with perceptions of rigid gender roles and is characterized by protective, patronizing attitudes toward women (Stermer and Burkley, 2015). However, a longitudinal study examining the influence of video game exposure on sexist beliefs and attitudes over a 3 year period found no evidence of a cultivation effect (Breuer et al., 2015). Further cross-sectional and longitudinal studies are therefore necessary.

In the present study, we were interested in the links between video games and sexist attitudes and we focused on the largest sample gathered to date on video games and sexism. We therefore hypothesized a positive relationship between overall video game exposure and the adherence to sexist attitudes (hypothesis 1). We investigated television as a comparison variable, because most of the available research on sexism and the media is based on TV content (Herrett-Skjellum and Allen, 1996; Morgan and Shanahan, 1997; Signorielli and Bacue, 1999; Coltrane and Messineo, 2000; Oppliger, 2007; Valls-Fernández and Martínez-Vicente, 2007; Nassif and Gunter, 2008; Eisend, 2010; Furnham and Paltzer, 2010; Paek et al., 2011; Scharrer, 2014; Matthes et al., 2016). As in other countries, French television depicts gender roles in very stereotypical ways, as indicated by the gender of the primary character, the associated product categories, the home or work setting and the working role of the primary character (Matthes et al., 2016).

It is therefore relevant to analyze simultaneously the respective links of these two kinds of media on sexism. It can be expected that the inherent interactivity of video game could prove to be more influential than television. In a study on media violence, it has been shown that children playing a violent video game were then more aggressive than those who merely watched the same violent video game in a passive way (Polman et al., 2008). We therefore expected that the link between sexist attitudes would be more strongly predicted by video game exposure than by TV exposure (hypothesis 2).

### Sexism as an Embedded Attitude

Although social psychological research has shown that sexist attitudes or behavior may be temporarily increased even by situational cues (Rudman and Phelan, 2010), sexism is generally embedded in a mesh of cultural beliefs and grounded in social and institutional practices. Long-term and institutional sources of sexism may interact with media influences on sexism. Media content may produce contrasted effects for different types of people or in different social context (Morgan et al., 2009). For example, although overall amount of television viewing was related to the endorsement of anti-egalitarian gender roles in a sample of Tokyo residents, the correlation was reversed in a subsample of politically conservative participants (Saito, 2007). In some cultural contexts, therefore, media exposure may counter gender stereotypes. For example, in Kuwait, exposure to US television appears as a significant predictor of less, and not more, gender stereotypical views (Abdulrahim et al., 2009). Religion embodies an important and ubiquitous influence on gender role representations (Morgan, 1987; Glick et al., 2002; Christopher and Mull, 2006; Maltby et al., 2010), and represents maybe the “single most important shaper for sex roles” (Wilson, 1978). We included religiosity in our model to compare the effects of video game to ideological commitments that consistently contribute to gender role beliefs. Many studies carried out in Europe and North America have shown that religiously affiliated people were more prone to support unequal gender roles and to consider that women are first of all housekeepers and mothers (Thornton et al., 1983; Wilcox and Jelen, 1991; Sherkat and Ellison, 1999; Ghazel, 2003; Voicu, 2009).

We therefore expected that religiosity would be positively related to sexism (hypothesis 3), but also that the link between video game exposure and sexism would be lower among high religious participants (hypothesis 4) following the above research showing that media can challenge rigid sex roles among traditional groups.

## Materials and Methods

### Sampling Procedure and Participants

The sample included 13520 participants aged 11–19 who were selected at school through stratified random sampling and is representative of the metropolitan areas of Grenoble and of Lyon, France. These two cities are the major municipalities of the second largest and wealthiest region of France. The sample population corresponded to urban France in terms of age, sex and school level. Students completed paper questionnaires in their classrooms. An assistant was present in the room and helped the participants upon request. The participation rate was slightly above 95%.

The sample included both public and private schools (with the rare exception of schools that have not contracted with the state) and all curricula, professional as well as the general (the latter being the pathway to college with the best students). National Institute of Statistics and Economic Studies indicated that in the year during which the survey was carried out, there were 99.4% of youth attending school at 12, declining to 89% 1 year prior to the baccalaureate^1^. After contacting all schools, half of them declined to participate (the participation was 46/67 in Grenoble, and 81/220 in Lyon, with a deficit in schools with reputation for excellence or “general curriculum” and the private schools). Classes were randomly selected in each school grade of every school. The sample is in line with the reference population in terms of percentage of students per grade, age, gender according to the available statistical elements provided by the education authorities.

### Ethics Statement

The study was approved by the National Commission on Computer and Freedoms (CNIL, decision n#2012-148). Ethics in social studies in France is entrusted to the CNIL which is a national independent body that has representatives in universities. The present study categorized as sensitive was therefore subjected to the most thorough examination by the CNIL (the full review level 3). It includes questionnaire, population, sampling process, data storage, and modalities for obtaining the person’s consent. Due to the juvenile status of the population in its largest part, and the fact that the survey took place at school, a multiple level consent process was designed. The consent process was reviewed by the CNIL and implemented along the lines of the CNIL. Consent of schools was obtained in writing both from school heads and provincial directorates of education (one for each sampling zone). Consent of parents was asked through a home liaison diary which is the usual means for communication between the school and the family. The parents had to return an approval/refusal written note (active consent). Children could orally refuse to participate to the survey in full, even if their parents had approved the survey. They also could omit responding to questions in the questionnaire. All written consent material is retained by school masters so that filed workers never learn any of the names of the students. Statistical material about approval/refusal in each classroom was communicated by the school head to the research team.

## Measures

All the measures were extracted from a larger survey based on the ISRD 2 Questionnaire (Enzman et al., 2010). Gender was coded as male = 1 (50.7%) or female = 2 (49.1%). Participants ages ranged between 11 and 19 (*M* = 14.5; *SD* = 1.36). As in other studies (e.g., Lien et al., 2001), fathers’ educational level was used as a proxy for socioeconomic status and was distributed into the following categories: (1) no degree, (2) lower than baccalaureate, (3) hold a baccalaureate, (4) higher than baccalaureate (*M* = 3,05; *SD* = 0.99). Albeit less than optimal, such a measure enables an approximation of socioeconomic status that is less subjected to biases or missing values than other estimations in the context of a school survey.

### Media Exposure

Media exposure was assessed by the following question: “How many hours per day did you spend on average last week, Monday to Friday playing computer or on the video game console?” and “How many hours per day did you spend on average last week, Monday to Friday watching TV, DVDs, films (including on the internet).” In order to ensure an appropriate distribution of the variables, we created 10 categories, from (1) 1 h per day to (10) 10 h or more per day. According to their estimations, participants spent 2.77 h per day watching TV (*SD* = 2.79) and 1.70 h per day playing video games (*SD* = 2.63).

Although this measure cannot estimate the quantity of sexist content adolescents were exposed to in video games and on television, women are frequently portrayed in sexist ways (e.g., Dietz, 1998; Beasley and Collins Standley, 2002; Burgess et al., 2007; Jansz and Martis, 2007; Downs and Smith, 2010), and this is the case in French media (Matthes et al., 2016). Time exposure to TV and video games may therefore be considered as an acceptable proxy for general exposure to sexist contents.

### Religiosity

Two measures of religiosity were included. Religious attendance was assessed by asking respondents to report the frequency they attended religious services, from *never* (1) to *every day* (6). The denominations were: Catholics: 27.4%; Muslims: 25.9%; Protestants: 1.9%; Jews: 0.9%; Christian orthodox: 0.7%; Anglicans: 0.1; others 3.8. Moreover, 39.4% declared no religion. Religious salience was measured by a 4-point scale of how important religion is in participant’s everyday life ranging from *not at all important* (1) to *very important* (4). The two measures being strongly related, we constructed a composite measure (Cronbach’s α = 0.72; *M* = 2.24; *SD* = 1.07).

### Sexism

Sexism was measured with a single-item question. Participants were asked the following Likert-type question: “A woman is made mainly for making and raising children,” from fully disagree (1) to fully agree (4), *M* = 1.53; *SD* = 0.9.

## Results

Preliminary analyses indicated that sexism was higher among males (*M* = 1.71, *SD* = 0.98 vs. *M* = 1.35, *SD* = 0.77, tcor(12922) = 23.59) and decreased as socioeconomic status increased (*r* = -0.13, *p* < 0.001). Religiosity was related to higher sexism (*r* = 0.23; *p* < 0.001). Television exposure (*r* = 0.08, *p* < 0.001) and video game exposure (*r* = 0.15, *p* < 0.001) were also both related to sexism at a bivariate level. The bivariate correlations between all the variables are presented in **Table 1**.^2^

**Table 1:** Correlations and descriptive statistics.

	TVexp.	Video G exp.	Religion	SES	Age	range	mean	*SD*
Sexism	0.08^∗∗∗^	0.15^∗∗∗^	0.23^∗∗∗^	-0.13^∗∗^	0.03^∗∗∗^	1–4	1.53	0.90
TVexp.		0.37^∗∗∗^	0.12^∗∗∗^	-0.14^∗∗∗^	0.01 ns	0–10	2.77	2.79
Video G exp.			0.08^∗∗^	-0.10^∗∗∗^	-0.05^∗∗∗^	0–10	1.7	2.63
Religiosity				-0.18^∗∗∗^	0.00	1–5	2.24	1.07
SES					-0.12^∗∗∗^	1–4	3.05	0.99
Age						11–19	14.5	1.36

*^∗∗^*p* < 0.01; ^∗∗∗^*p* < 0.001.*

In order to test our primary hypotheses, a stepwise multiple regression was performed entering age, gender, socioeconomic level, religiosity, TV exposure and video games exposure in a first step. We also introduced in a second step interactions between video games exposure and age, gender, socioeconomic level, and religiosity. As expected, video games exposure was related to sexism (β = 0.07, *t* = 8.10, *p* < 0.001). This was also the case of religiosity (β = 0.20, *t* = 23.92, *p* < 0.001). Sexism was more endorsed by males (β = -0.18, *t* = -0.20.46, *p* < 0.001). Moreover, higher socioeconomic level predicted lower sexism (β = -0.09, *t* = -0.10,36, *p* < 0.001). After controlling for all these variables, television exposure was unrelated to sexism (β = 0.015, *t* = 1.66, *p* < 0.10). No interaction effects were observed. The overall determination coefficient was *R*^2^ = 0.104, *p* < 0.001.

## Discussion

In this cross-sectional study based on a representative sample of 13520 French youth aged 11–19 years, we showed that general video game exposure was significantly related to sexism, irrespective of gender, age, socioeconomic status, and religion. We also observed that general television exposure was unrelated to sexism after each of the variables were controlled. Of course, the usual critiques aimed at cross-sectional surveys fully apply here, particularly the limitations in terms of assessing causality. It may be that individuals with sexist orientations spend more time playing videogames (Fox and Tang, 2016). In order to address causality issue, future experimental studies could be based on the same protocol as Polman et al. (2008), who showed that people were more influenced by the content of a scene where they were actively playing themselves the game compared to a condition in which they merely passively watched the screen with the same contents.

It also should be noted that the determination coefficient was small, which suggest that many important correlates of sexism were not measured in our survey. For example, it has been shown that sexism is related to gender inequality (Glick et al., 2004; Napier et al., 2010), social dominance, right-wing authoritarianism (Christopher and Mull, 2006), and conservative values (Mikolajczak and Pietrzak, 2014). The mere introduction of such social psychological variables may affect the predictive value of general video game exposure.

Another limitation is the underrepresentation of some categories of schools in our sample, with a deficit in schools with reputation for excellence or “general curriculum” and the private schools. Studies suggest that sexism may be endorsed more strongly in lower social classes (Gianettoni and Simon-Vermot, 2010), which are usually less represented in such schools. Such a sampling feature may have affected the results of the survey.

It should also be mentioned that the use of a single-item scale to assess sexism was less than optimal. This item was actually the only measure of sexism in the ISRD2 survey, and represents a very specific dimension of sexism (motherhood or domesticity) that hardly captures all possible forms of sexist thinking. As previously indicated, experimental studies showed that playing sexualized video games for a few minutes promoted gender stereotypes (Behm-Morawitz and Mastro, 2009; Yao et al., 2010), increased hostile sexism (Fox and Bailenson, 2009), and men’s acceptance of sexual harassment (Dill et al., 2008). Future survey studies should include measures of objectification and standard measures of benevolent and hostile sexism (Glick and Fiske, 1996). Finally, we used a general measure of TV and video game experience rather than measuring exposure to specifically sexist portrayals.

A strength of this study is the multivariate approach, including other known predictors of sexist attitudes. Video game exposure was a significant factor even after controlling for age, sex, socioeconomic level, television exposure, and religiosity, which is considered a particularly strong determinant of sexism (Morgan, 1987; Glick et al., 2002; Christopher and Mull, 2006; Maltby et al., 2010). The link between religion and sexism was, however, three times higher than the links between video games and sexism. Contrary to our expectations, the link between video games exposure and sexism was not moderated by religion in this sample. The importance of religiosity in the prediction of sexism would justify a fine-grained approach in future research based on religious orientations (Batson et al., 1993). Interestingly, participants with a higher socioeconomic level endorsed less sexism. This is consistent with a behavioral study carried out in an online first shooters video game showing that low status players were more hostile toward a female teammate, while high status players were more positive toward her (Kasumovic and Kuznekoff, 2015).

The causal link between video game exposure and sexism in action deserves to be confirmed by experimental studies. This is especially the case for sexist behaviors, which have been under-studied (Stermer and Burkley, 2012). If confirmed, however, the video game industry may find it appropriate to encourage an evolution in the way women are represented, because sexism on screen can have consequences which are not limited to the virtual world. Today, 48% of video game players are female (Entertainment Software Association, 2014), and in addition to the development of sexist attitudes, the repeated exposure to biased female models on games produces body dissatisfaction among women (Holmstrom, 2004), self-objectification (Fox et al., 2015) and eating disorders (Grabe et al., 2008).

## Conclusion

We showed for the first time in a large representative sample that video game exposure was related to sexism, controlling for television exposure, religiosity, and other relevant factors. Our results suggest that a traditional source of influence (religiosity) as well as new digital media may share some similar features on sexism.

## Author Contributions

SR designed and directed the survey. LB analyzed the data and wrote the paper, helped by ES, CB, DG, and SR. The whole co-authors contributed to the final draft.

## Conflict of Interest Statement

The authors declare that the research was conducted in the absence of any commercial or financial relationships that could be construed as a potential conflict of interest. The reviewer AC and the handling Editor declared their shared affiliation, and the handling Editor states that the process nevertheless met the standards of a fair and objective review.

## Footnotes

1 Baccalaureate is a school-leaving examination leading to university entrance qualification in the French school system. In France, school is compulsory until the age of 16.

2 We performed bivariate analysis among the two largest religious groups: Catholics and Muslims. The correlation between video game exposure and sexism was, respectively, 0.18 and 0.11 (*p* < 0.001 for each). The correlation between television exposure and sexism was 0.08 and 0. 01 (ns). This latest result is consistent with studies showing that television impact on sexism is not homogeneous among social subgroups (Saito, 2007; Abdulrahim et al., 2009).
